# Removal of intramedullary lipoma by endoscopic technique: a case report

**DOI:** 10.3389/fsurg.2026.1784777

**Published:** 2026-04-13

**Authors:** Yanwang Niu, Jingchao Wei, Yusong Guo, Wenyi Li, Yuxin Meng, Shangju Gao

**Affiliations:** 1 Hebei North University, Zhangjiakou, Hebei, China; 2 Department of Orthopedics, Hebei General Hospital, Shijiazhuang, Hebei, China

**Keywords:** case report, endoscopic technique, intramedullary lipoma, intraspinal tumor, minimally invasive

## Abstract

The lipoma is a common benign tumor of the subcutaneous body, and it is commonly found on the surface of the skin. But, it can also involve any tissue or organ. Intraspinal lipomas are rare, which account for less than 1% of intraspinal tumors. Among these, intramedullary lipoma is an even rarer condition, compared to extramedullary subdural or epidural types. Intraspinal tumors were removed by the open surgery in common, due to its adequate intraoperative visualization and complete resection. But for intraspinal lipomas, it does not exhibit implantation metastasis, making minimally invasive spinal endoscopic surgery considered as an option. We performed a resection of a intramedullary lipoma, and the surgery was conducted under the guidance of spinal endoscopy. Compared with open surgery, minimally invasive spinal endoscopic surgery may be more advantageous in reducing soft tissue injury and bony structure destruction, allowing patients to achieve rapid recovery. The symptoms of numbness in the left limb and unsteady gait of the patient completely disappeared after the operation. It should be noted that continuous bleeding during the operation exacerbates the complexity of the surgery. When removing lipomas, patience and meticulousness are crucial for the operation.

## Introduction

The lipoma is a common benign tumor of the subcutaneous body, arising from mesenchymal cells (1). However, it can also involve any tissue or organ, and its pathogenesis remains unclear (2). Sometimes, it appears in critical positions, which can cause serious symptoms, such as in the spinal cord. Intraspinal lipomas are rare, which account for less than 1% of intraspinal tumors (1). Among these, intramedullary lipoma is an even rarer condition, compared to extramedullary subdural or epidural types. According to traditional intraspinal localization methods, they are typically classified into: intramedullary lipoma, extramedullary subdural lipoma, and epidural lipoma. As with other intraspinal tumors, symptoms were usually indefinite in the early stage due to the mild compression from the small tumor. When the tumor grew to further compress the specific spinal cord regions, the corresponding neurological symptoms emerge. Surgical intervention is necessary for symptomatic patients to prevent worse neurological damage (3).

Intraspinal tumors (lipoma) were removed by the open surgery in common, due to its adequate intraoperative visualization and complete resection. But it does not exhibit implantation metastasis, making minimally invasive spinal endoscopic surgery considered as an option. Although traditional open spinal surgery can remove tumors with high precision while minimizing the risk of nerve damage, minimally invasive spinal endoscopic surgery may be more advantageous in reducing soft tissue injury and bony structure destruction (4, 5). Growing evidence indicates that minimally invasive surgery is comparable to open surgery in terms of extent of resection and the restoration of neurological function, while being associated with fewer complications and lower medical costs (5, 6).

We described a case of intramedullary lipoma successfully removed via minimally invasive spinal endoscopic surgery. Significant neurological function improvement was observed immediately after surgery. It indicates that minimally invasive surgery can achieve satisfactory clinical outcomes for non-implantative intraspinal tumors, which provided a valuable reference for surgical decision-making process.

## Case presentation

**Figure 1:**
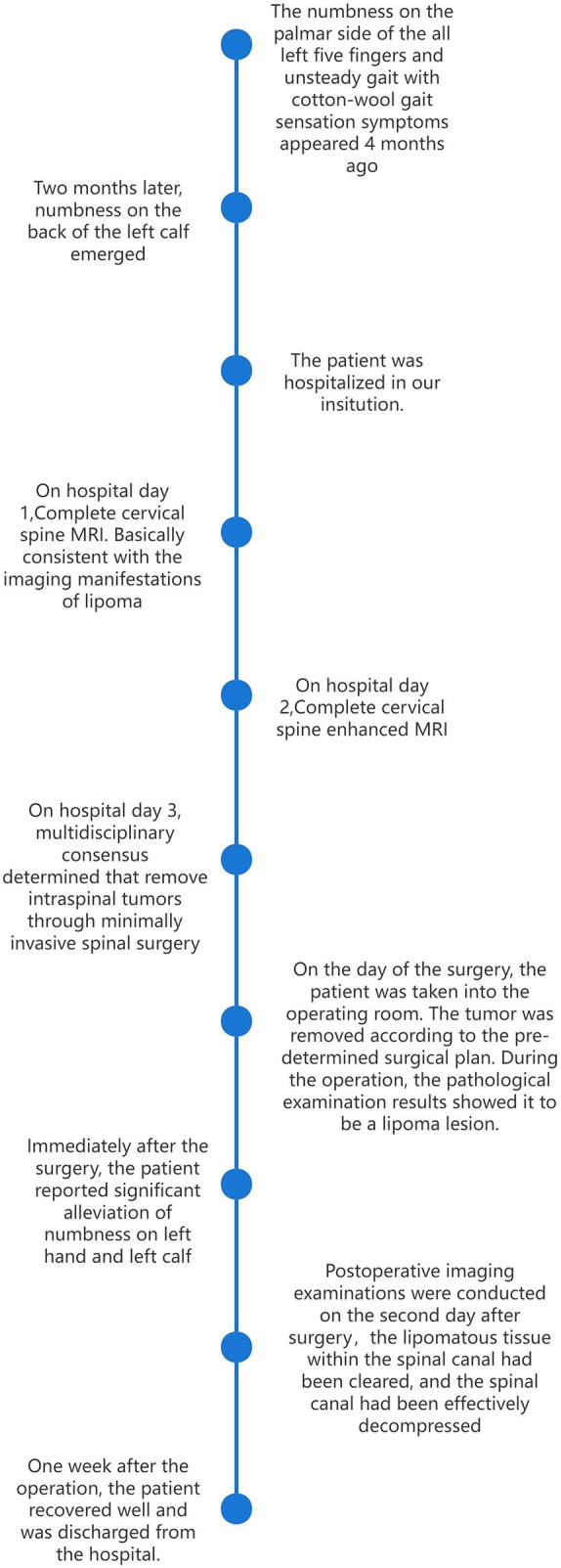
Patient visit flowchart.

This 43-year-old male patient presented with a 6-month history of left hand numbness and unsteady gait (Figure 1). The symptoms began with numbness on the palmar side of the all left five fingers and unsteady gait with cotton-wool gait sensation. Two months later, numbness on the back of the left calf emerged. Physical examination revealed active bilateral knee-jerk reflexs and grade IV muscle strength in the left biceps brachii, wrist flexors, and wrist extensors on admission.

**Figure 2:**
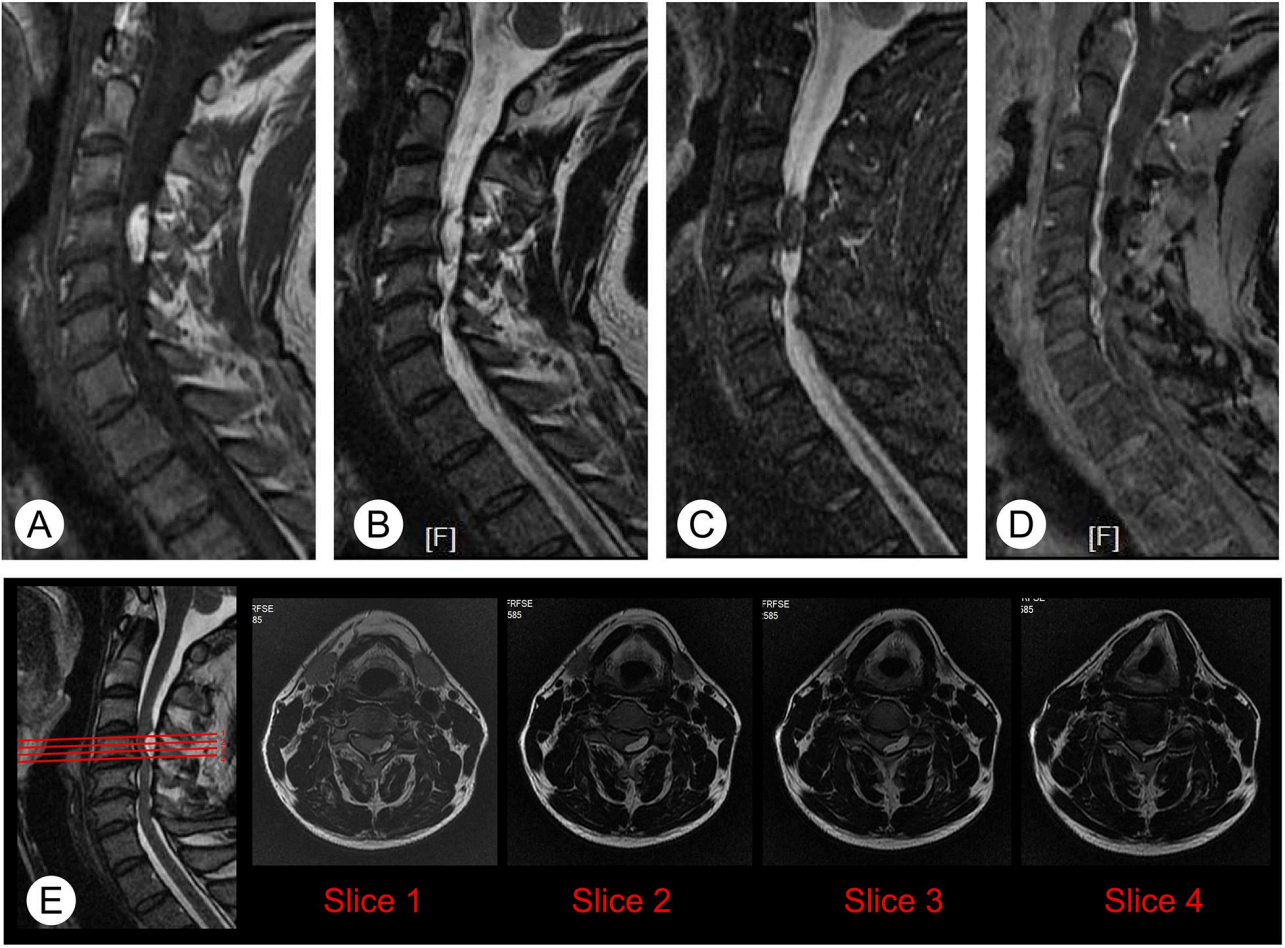
Magnetic resonance images of the cervical spine before surgery **(A,B)** T1- and T2-weighted images revealed a hyperintense lesion located posterior to the C4 vertebral body. **(C)** Fat-suppressed images showed the lesion appears hypointense with several internal linear signal voids. **(D)** Contrast-enhanced T1-weighted images similarly demonstrate hypointense of the lesion. **(E)** Axial T2-weighted images (slices 1−4) and their location image suggested the lipoma located entirely posterior to the C4 vertebral body.

MRI findings are consistent with typical features of lipoma, meaning that the lesion showed hyperintense on both T1- and T2-weighted images (Figures 2A,B), with hypointense on fat-suppressed sequences (Figure 2C). In addition, the contrast-enhanced MRI was performed to aid the diagnosis and no enhanced signal was observed within the lesion (Figure 2D). The axial T2-weighted images (Figure 2E) were carefully observed, and slices 1–4 suggested the lipoma located entirely posterior to the C4 vertebral body.

## Operation procedure

The patient underwent spinal canal decompression and intramedullary lipoma resection via posterior cervical endoscopic technique. Under general anesthesia, the patient was in prone with the flexural cervical spine. The left C4 lamina was localized with C-arm fluoroscopy guidance, and marked on the skin. After the soft tissue trocars were inserted in sequence via a 0.7 cm skin incision, the working cannula was put in place. A trephine was inserted to leave a slight imprint on the left C4 lamina as a mark. After the above preparation, the spinal endoscope was inserted through the working cannula, and start the endoscopic operation.

**Figure 3:**
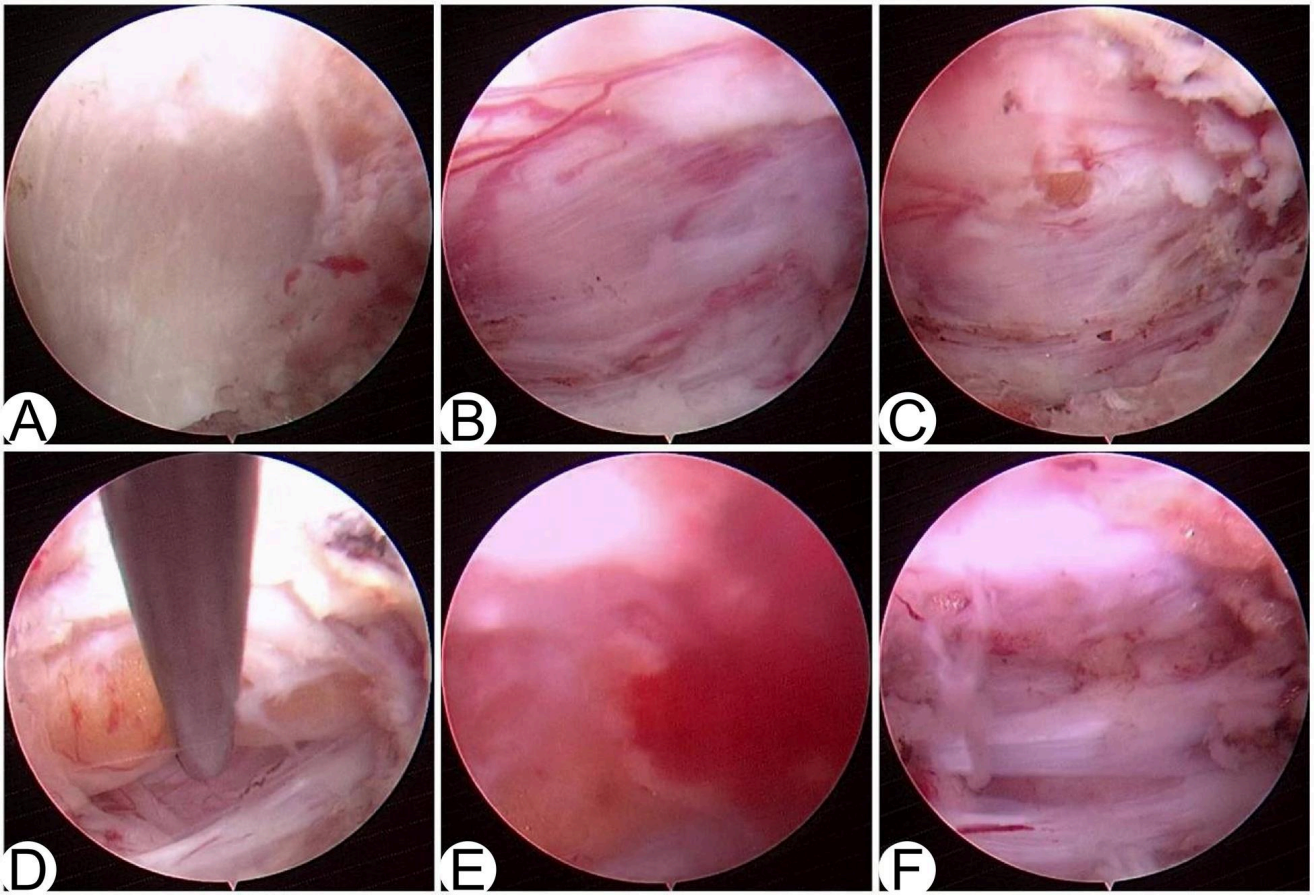
Endoscopic view in minimally invasive spine endoscopic surgery **(A)** the exposure of the left C4 lamina following careful dissection of surrounding soft tissues. **(B)** The dural sac exposed after adequate removal of the left C4 lamina with a high-speed burr. **(C)** Yellow lipomatous tissue was observed beneath the dura. **(D)** Attempt to separate the lipoma using a nerve dissector. **(E)** Intraoperative bleeding occurred during partial removal of the lipoma with pituitary forceps. **(F)** The nerve root clearly visualized after complete decompression and there is a small amount of lipomatous tissue remaining.

Before laminectomy, surrounding soft tissues was removed using a pituitary rongeur and radiofrequency to expose the left C4 lamina (Figure 3A). Along the trephine markings of the C4 lamina, the marking area and its cranial and caudal margins were meticulously removed by a high-speed burr, and the ligamentum flavum was fully exposed. Subsequently, Kerrison rongeurs were used to resect the ligamentum flavum, in order to expose the dural sac. The pulsating dural sac was observed (Figure 3B, and opened meticulously with the basket forcep.

Through a small incision, yellow lipomatous tissue was observed beneath the dura (Figure 3C), suggesting the intramedullary lipoma. The dural opening was enlarged by alternating the use of a nerve hook, hook knife, and rongeur, during which the yellow lipomatous tissue was mobilized together with the dura. Using the nerve hook, the ventral aspect of the lipomatous tissue was explored in an attempt to separate it from the spinal cord (Figure 3D). A lipomatous mass was visualized overlying the dorsal surface of the spinal cord, and the mass tissue was progressively excised with a pituitary rongeur. The lipoma continued to bleed intraoperatively and obscured the endoscopic field of view (Figure 3E), indicating a rich internal vascular supply. Intraoperative bleeding affected endoscopic operation so severely thatimpaired endoscopic visualization. Therefore, meticulous hemostasis was essential. Tumor resection was carried out with simultaneous coagulation, where patience and precision were critical to surgical success. After most of the resection was complete, the remaining tumor tissue exhibited an indistinct margin with the left C4 nerve root (Figure 3F). To avoid iatrogenic injury to nerve roots, a small portion of the lipomatous tissue adjacent to the nerve root was deliberately preserved.

Remove the spinal endoscope and working cannula step by step. Finally, the surgical incision was sutured. The tissue specimen was sent for pathological examination, and the result confirmed the diagnosis of lipoma.

## Postoperative assessments

Immediately after the surgery, the patient reported significant alleviation of numbness on left hand and left calf. He was able to walk by himself with a cervical collar on the first day after the surgery, and cotton-wool gait sensation disappeared completely. Muscle strength was reassessed at discharge, and remained consistent with preoperative levels.

**Figure 4:**
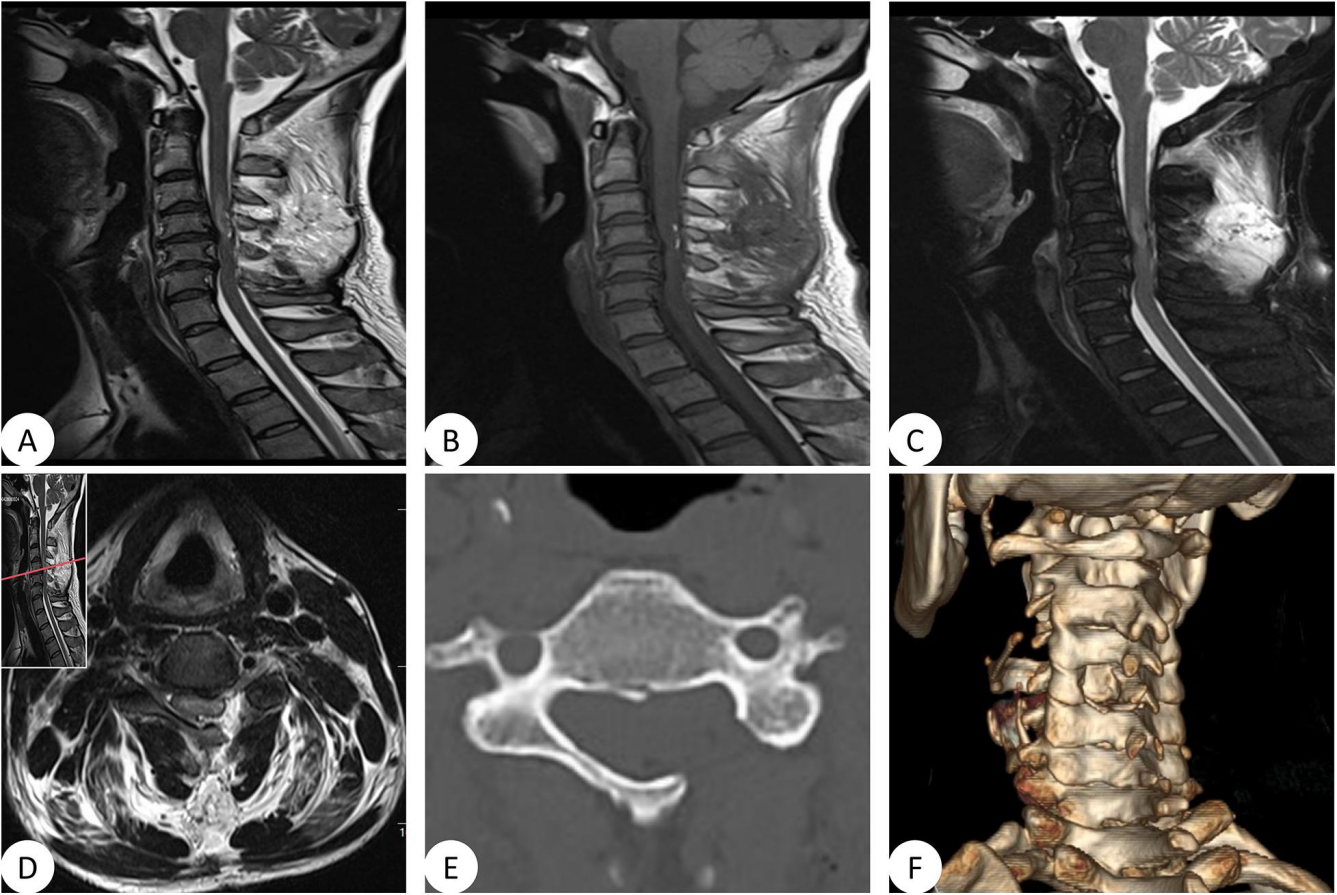
Magnetic resonance images of the cervical spine after surgery. **(A–C)** T1-weighted, T2-weighted, and fat-suppressed images demonstrated subtotal resection of the lipomatous lesion, with a small residual component remaining. **(D)** Axial T2-weighted images confirmed complete decompression. Computed tomography and three-dimensional reconstruction of the cervical spine after surgery. **(E)** Axial images showed that the left C4 lamina was removed. **(F)** Three-dimensional reconstruction showed that left C4 lamina was removed totally.

Postoperative imaging examinations were conducted on the second day after surgery. The left lamina of C4 was removed completely was observed on the postoperative CT scan (Figures 4E,F). On the postoperative MRI, it was noted that the lipomatous tissue within the spinal canal had been mostly cleared, due to the severe adhesion between the lipoma tissue and the spinal cord during the operation, in order to protect the patient's nerve function to the greatest extent, a small portion of the lipoma tissue with severe adhesion to the spinal cord was left at the posterior part of the cervical vertebra, and the majority of the tumor tissue was removed, and the spinal canal had been effectively decompressed (Figure 4A–D).

## Discussion

Intramedullary lipoma has an incidence of less than 1% (1). However, the reported incidence rates vary between Chinese and international studies (3, 5). Additionally, the disease tends to affect patients at a relatively young age, with two-thirds of cases presenting before the age of 30 (6). It typically has an insidious onset and a prolonged course. This condition may occur as an isolated finding or coexist with spinal cord and vertebral deformities. This condition can occur at any segment of the spinal cord. However, the pathogenesis of this condition remains unclear. A hypothesis regarding about the disease was put forward that during spinal neural tube closure, a portion of displaced adipose tissue becomes incorporated (2).

In the auxiliary examinations for lipoma, MRI demonstrates a high diagnostic value (7, 8). In the fat-suppression sequence, the high-signal-intensity fatty areas within the lesion are suppressed, demonstrating extremely low or no signal intensity, which provides diagnostic utility. However, the definitive characterization of the tumor still relies on pathological examination. When the tumor invades the vertebral body, CT scan is essential and important to aid in the differentiation of epidural lipoma (9).

Preventive surgery for asymptomatic patients was recommended (9). Even if patients undergo partial tumor resection, adjuvant radiotherapy and chemotherapy are unnecessary for benign tumors (7, 10). It is preferable to perform adequate decompression rather than attempting complete resection to achieve better clinical outcomes before severe neurological deficit (10, 11). Notably, the application of neurophysiological monitoring in spinal tumor surgery has been proven to reduce intraoperative nerve damage (12). Therefore, we did not completely resect the lesion tissue enveloping the nerve roots to maximize neural function preservation. In accordance with expectation, the symptoms of limb numbness and unsteady gait completely resolved postoperatively.

Shin DA et al. reported patients underwent posterior cervical open surgery, and their symptoms of quadriparesis with numbness significantly improved postoperatively (3). Compared with traditional open surgery, minimally invasive spinal surgery has also achieved promising outcomes in addressing intraspinal lipoma diseases (6, 12). Wong AP et al. Reported traditional open surgery is useful in achieving removing lipoma tissue and minimal long-term neurological impairment. On the other hand, minimally invasive surgery may be more beneficial in reducing the impact on soft tissue and midline structures, as well as reducing the duration of in-patient stays (13). However there remains considerable variation in the selection of the optimal surgical approach. We considered that lipoma is a benign tumor, which has a favorable prognosis and a low recurrence rate, and minimally invasive spinal surgery minimized tissue trauma and the patient can achieve fast recovery. But it should be noted that continuous bleeding during the operation exacerbates the complexity of the surgery. When removing lipomas, patience and meticulousness are crucial for the operation.

Primary spinal cord tumors represent 2%–4% of all neoplasms of the CNS. Primary spinal cord tumors are anatomically separable into two broad categories: intradural intramedullary and intradural extramedullary. Intramedullary tumors are comprised predominantly of gliomas (infiltrative astrocytomas and ependymomas). Resective surgery can usually be accomplished with spinal ependymomas owing to separation of tumor from spinal cord and, when complete, require no further therapy (14). However, astrocytomas infiltrate the spinal cord and complete resection is rare. Radiotherapy is reserved for malignant variants and recurrent gliomas, whereas chemotherapy is administered for recurrent primary spinal cord tumors without surgical or radiotherapy options. Early recognition of the signs and symptoms related to primary spinal cord tumors facilitates timely discovery, treatment, potentially minimizes neurologic morbidity, and may improve outcome (15).

During the preoperative planning, given that lipoma is a benign lesion with no potential for metastasis, a spinal endoscopic approach was attempted. Pruthi N et al. (3) reported that patients with spinal lipomas uniformly underwent surgical resection, and long-term follow-up revealed no evidence of tumor recurrence. However, in cases with a high suspicion of malignancy, minimally invasive spinal endoscopy is not advisable, as tumor cells may disseminate within the aqueous medium. Furthermore, the present patient has not yet undergone long-term follow-up, and whether postoperative recurrence will occur remains to be determined.

## Conclusion

This case of intramedullary lipoma successfully removed via minimally invasive spinal endoscopic surgery, and achieved satisfactory clinical outcomes. It indicates that minimally invasive surgery was an option for non-implantative intraspinal tumors.

## Data Availability

The original contributions presented in the study are included in the article/Supplementary Material, further inquiries can be directed to the corresponding author.
